# The validation of a simple and instrument‐free technique to measure the depth of the natal cleft (a cohort study)

**DOI:** 10.1111/codi.70041

**Published:** 2025-03-04

**Authors:** Dotun Ojo, Paul Bassett, Gregory Thomas, Asha Senapati

**Affiliations:** ^1^ St Mark's Hospital London UK; ^2^ Imperial College London UK; ^3^ Queen Alexandra Hospital Portsmouth UK

**Keywords:** depth of natal cleft, instrument‐free, natal cleft, pilonidal disease

## Abstract

**Aim:**

Pilonidal disease is a benign condition mainly affecting the young population. In recent literature, the depth of the natal cleft is thought to impact postoperative outcomes including wound complications and rate of recurrence. Currently there is no agreed method for measuring the depth of the natal cleft; this study proposes a novel and instrument‐free technique that can be used as a research tool to measure the depth.

**Method:**

This is a single centre study performed at St Mark's Hospital in London, with 63 participants volunteering to take part. Blinded repeated measurements of the natal cleft were taken by two separate clinicians to assess for inter‐observer and intra‐observer variation.

**Results:**

Agreement was measured and assessed by the Bland–Altman limits of agreement method and intra‐class correlation (ICC). The mean difference between repeat measurements for both inter‐observer and intra‐observer were close to zero (−0.06 and −0.11 respectively). ICC analysis suggested a value of 0.91 for inter‐observer agreement, signifying very good agreement between clinicians. Moderate intra‐observer agreement was observed, with an ICC value of 0.74.

**Conclusion:**

This study offers an alternative simple validated technique to measure the depth of the natal cleft, with good observer agreement. We propose that future studies aiming to analyse the depth of the natal cleft and its relationship with severity and postoperative outcomes use this technique.

**What does this paper add to the literature?**

This study provides a simple and instrument‐free technique for measuring the natal cleft in a standardised manner that is easily replicable. We believe that future research intent on researching depth of the natal cleft and its relationship with severity and postoperative outcomes consider the use of this technique.

## INTRODUCTION

Pilonidal disease is a benign condition mainly affecting the young population [1]. Risk factors including smoking, obesity, long duration of sitting and trauma traditionally have been attributed to pilonidal disease [2]. The depth of the intergluteal groove, or natal cleft, which is linked with weight and obesity, is thought be greater in those with the disease and also to contribute to the risk of postoperative wound complications. It has been suggested that the natal cleft may flatten with age and this may be the reason pilonidal disease occurs less frequently as people get older. It is also postulated that recurrence after surgery is more likely in patients with a deeper natal cleft, which allows debris to collect [3, 4], triggering the cycle of infection. Off‐midline closure techniques such as the Karydakis, Limberg and Bascom's cleft lift [5, 6, 7] aim to flatten the natal cleft as part of the procedure [8], and its low recurrence rate when compared to midline closure could be attributed to this [9]. A previous study had demonstrated how a technique similar to a Bascom's cleft lift flattened the natal cleft with a mean reduction of depth of near 2 cm [10]. Ultimately, there are many techniques used to treat pilonidal disease as was demonstrated in a recent PITSTOP study [11, 12]; decision making regarding the best technique may be helped in the future by knowing the depth of the natal cleft.

There have been recent studies suggesting a relationship between the depth of the cleft and postoperative complications. Kasim et al. concluded that the depth of the natal cleft was an important factor, demonstrating a positive correlation with depth and time of healing [13].

In the literature there is no agreed method for measuring the depth of the natal cleft. Previously Akinci et al. developed a specific calliper tool for this purpose [3]. However, if the site of the measurement is not specified, the measurement will vary as the natal cleft depth is not uniform. The cleft increases in depth cranially to caudally, so the depth will vary depending on the precise site at which it is measured.

We propose a simple and instrument‐free research tool for measuring the natal cleft in a standardised manner that we believe could be used for all patients to stratify severity and inform decision making about treatment for pilonidal disease. It may also allow comparisons between studies and to assess risk factors accurately in relation to the natal cleft.

This study describes the technique and aims to validate this technique by demonstrating little discrepancy between intra‐ and inter‐observer variation in obtained measurements.

## METHODS

This is a single centre study performed after approval from the national research ethics committee (IRAS ID 291048). Eligibility criteria included patients aged 18 or older, and there were no gender or socioeconomic restrictions. Participants unwilling or unable to provide informed consent were not included within the study.

### Sample size

A power calculation was performed in order to establish the required number of patients to study. A sample size was calculated based on achieving a sufficiently accurate estimate of the agreement between repeat measurements of natal cleft depth. Agreement was measured and assessed by the Bland–Altman limits of agreement method and intra‐class correlation (ICC). A proposed acceptable margin of difference of 0.5 cm between repeat measurements was considered acceptable. Assuming that agreement was at or around this margin, a standard deviation of the differences between repeat measurements was assumed of 0.25 cm. Bland–Altman limits were quantified to within ±0.125 cm, which was deemed sufficiently accurate. With a 95% confidence level, a calculation of at least 46 patients was deemed to be required for the study.

### Technique

**FIGURE 1:**
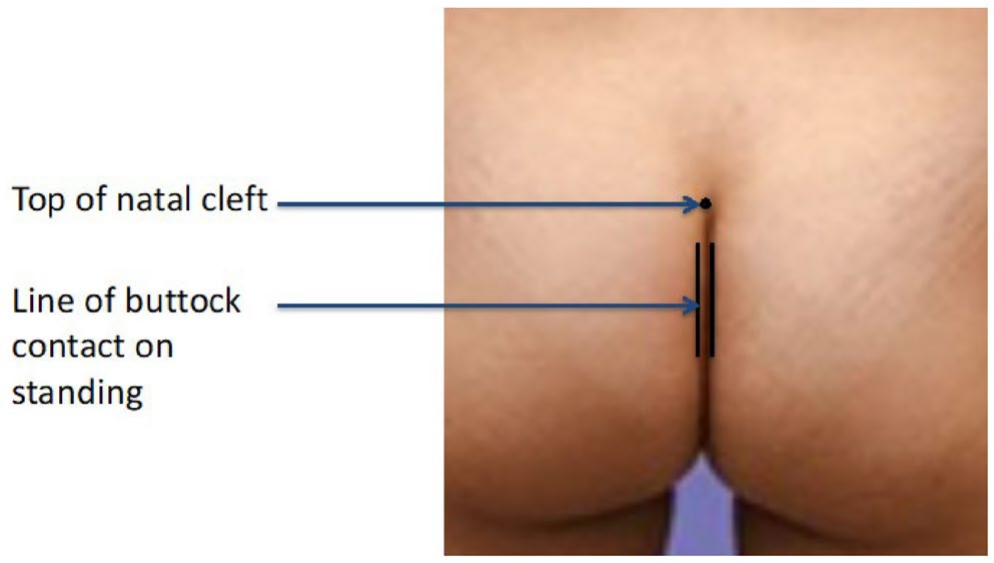
Marking the natal cleft.

**FIGURE 2:**
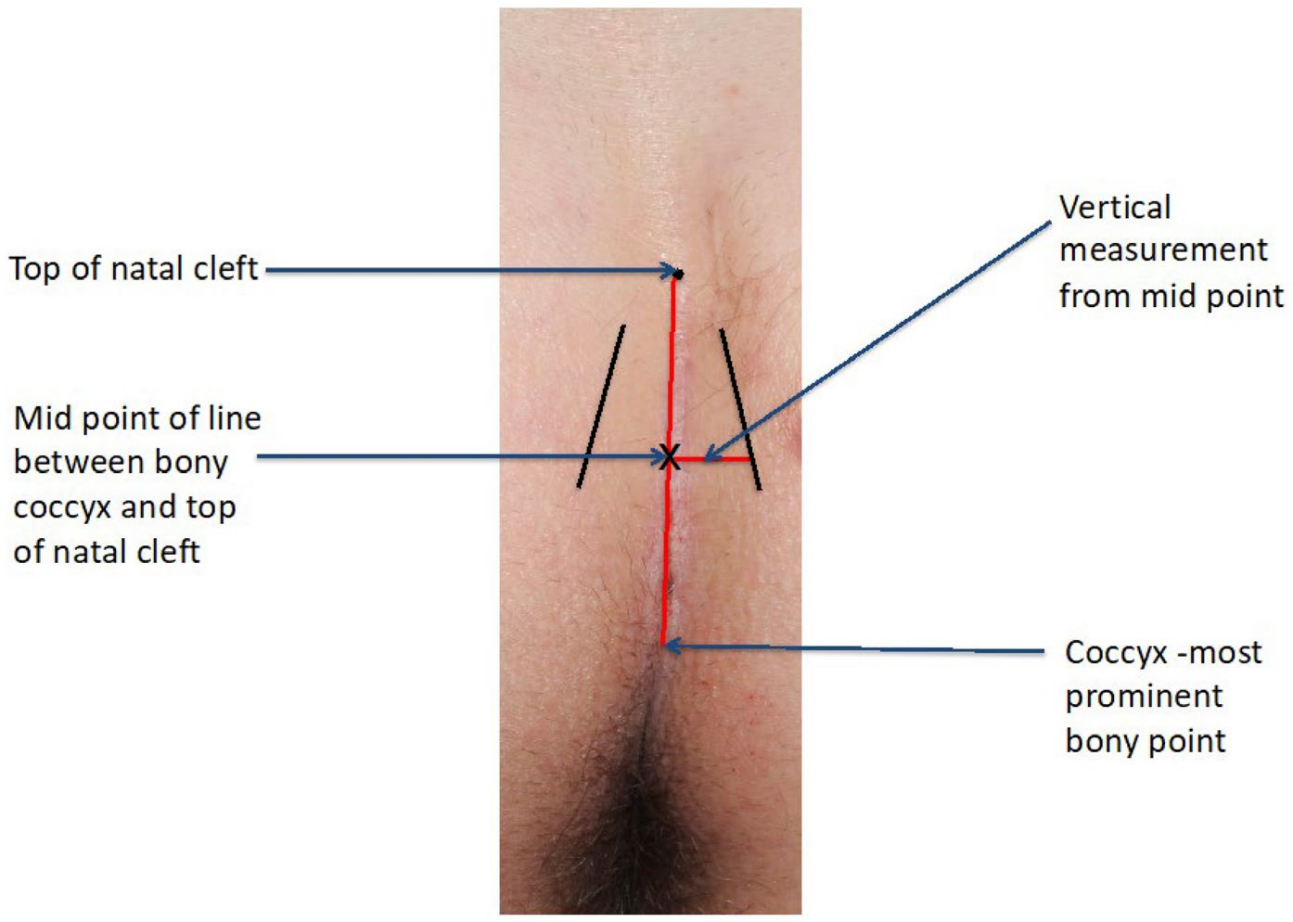
Measuring the depth of the cleft.

1. The patient is asked to stand upright facing away from the clinician. A fine marker pen is used to place a dot at the top of the natal cleft, which is easily identified. Two vertical lines are made on each buttock where both cheeks meet (Figure 1). This should be done whilst the patient is standing as buttocks fall apart once the patient lies prone.
2. In clinic or in theatre before using tape to separate the buttocks, the most caudal part of the coccyx is palpated at the lower end of the natal cleft. The distance is measured between this and the marked dot. This distance (the length of the cleft) is divided by two to identify the midpoint of the natal cleft.
3. The depth of the cleft is measured as the distance between this midpoint and either of the lines on the buttock cheeks, and is rounded to the nearest 0.25 cm (Figure 2). The measurements are taken with the patient lying prone.

Two clinicians (one expert and one trainee) were involved in this study and were trained in the technique of taking measurements of the midpoint of the natal cleft in order to obtain independent measurements. Patients attending the pilonidal clinic were previously sent a patient information sheet explaining the purpose of the study and the technique to be used. Consent was then taken in clinic if patients were willing to participate. Participants could withdraw at any point within the study if they decided no longer to participate.

The two blinded measurements were taken either in clinic or preoperatively by clinicians 1 and 2 respectively to obtain inter‐observer readings. If clinician 1 had taken a previous measurement in clinic, a further measurement was taken immediately preoperatively in order to obtain intra‐observer readings. Assessors were blinded to measurements by the other assessor and to initial measurements made by themselves.

## RESULTS

A total of 63 (44 male) patients were recruited to the study between January 2022 and March 2023. The average age of the cohort was 31 years (range 20–56). Sixty‐three pairs of measurements were available to assess inter‐observer agreement and a total of 38 pairs of measurements were available for assessment of intra‐observer agreement.

**FIGURE 3:**
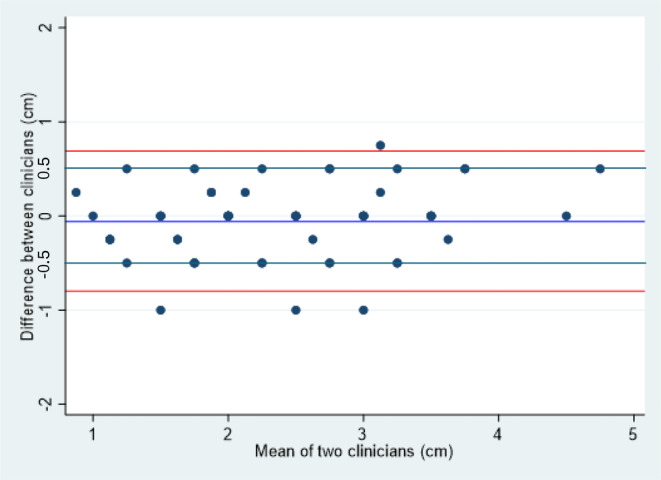
Bland–Altman limits for inter‐observer agreement.

**FIGURE 4:**
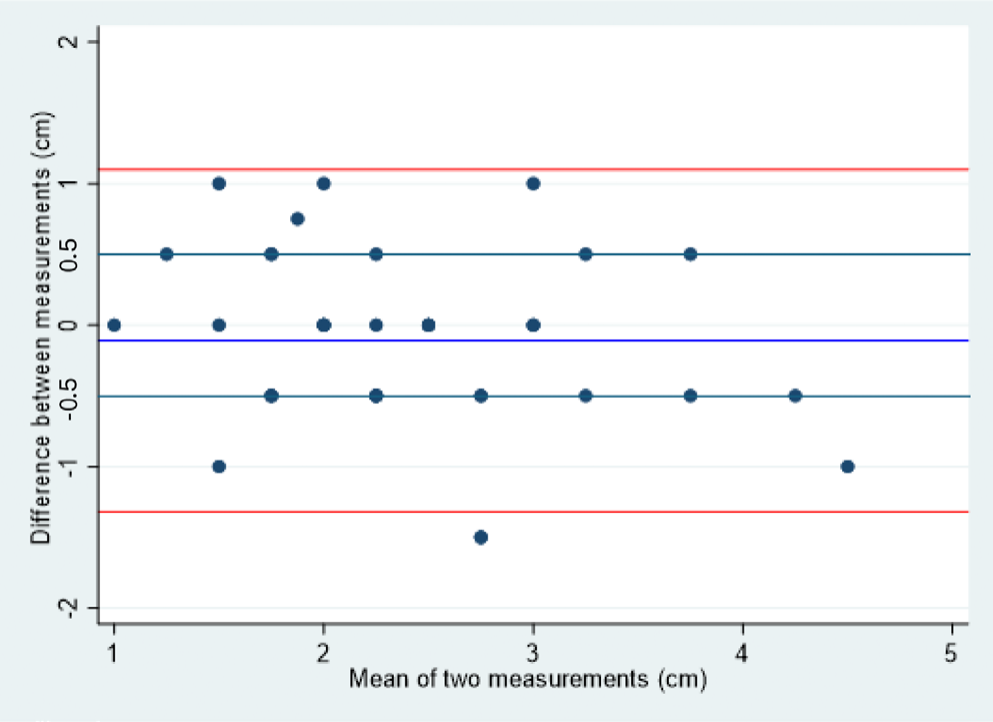
Bland–Altman limits for intra‐observer agreement (clinician 1).

**FIGURE 5:**
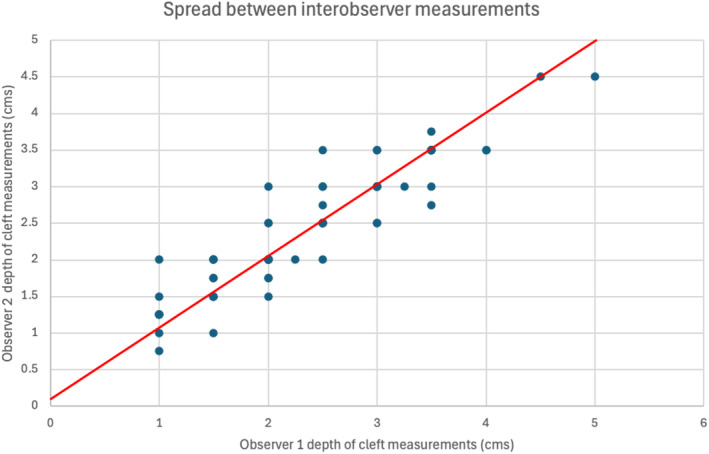
Demonstrating correlation between inter‐observer measurements; Pearson's correlation coefficient 0.91.

**FIGURE 6:**
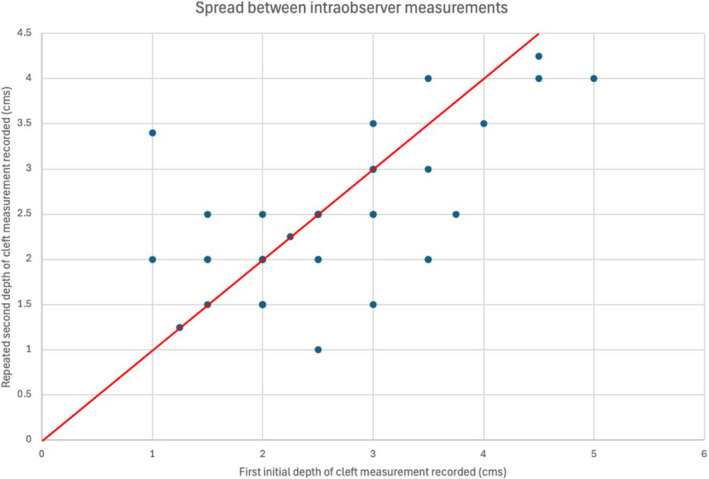
Demonstrating correlation between intra‐observer measurements; Pearson's correlation coefficient of 0.74.

Table 1 shows the results, with an ICC of 0.91 for inter‐observer variation and 0.74 for intra‐observer variation. These results are displayed graphically in a Bland–Altman plots, plotting the differences between pairs of measurements against the average of the two scores (Figures 3 and 4). Figure 5 demonstrates the spread of results for inter‐observer agreement whilst Figure 6 demonstrates results for intra‐observer variation.

**TABLE 1:** Inter‐ and intra‐observer agreement (Bland–Altman method).

Agreement	*n* pairs	Mean difference	SD difference	95% Bland–Altman limits	ICC (95% CI)
Inter‐observer	63	−0.06	0.38	−0.80, 0.69	0.91 (0.86, 0.95)
Intra‐observer	38	−0.11	0.62	−1.32, 1.10	0.74 (0.55, 0.85)

*Note*: Inter‐observer: Using data from measurements 1 and 2 only; differences calculated as clinician 1 minus clinician 2. Intra‐observer: Using data from measurements 1 and 3 only; differences calculated as measurement 3 minus measurement 1.

Abbreviation: ICC, intra‐class correlation.

## DISCUSSION

The role of the depth of the natal cleft in pilonidal disease is a topic often explored by surgeons with a specialist interest in pilonidal disease. The technique we have proposed is standardised to allow any clinician to measure the depth of the natal cleft in a uniform way using anatomical landmarks. Previous studies measuring the depth of the natal cleft have developed a specific tool to perform measurements. We have offered a technique that requires no instrumentation and guides a non‐expert with a step‐by‐step standardised approach to obtain measurements at a specified point.

The ICC of 0.91 suggests that there is very good agreement between pairs of measurements taken by two clinicians at a single given time. Analysis of intra‐observer measurements suggests that the level of agreement is somewhat poorer (ICC of 0.74), with only moderate agreement between these pairs of measurements. One reason for this may be the amount of time that elapsed between the two intra‐observer measurements taken due to the length of waiting lists at the time. The mean interval between readings was 21.7 weeks (range 1–110). This study was conducted during the latter part of coronavirus restrictions when there were prolonged periods of time between patients first attending clinic and having surgery. It is possible that patients may have gained or lost weight during this time. However, the majority of the measurements in both the inter‐ and intra‐observer studies lie within 0.5 cm of each other. This technique could be used in future studies potentially to correlate the depth of the natal cleft with severity of the disease or possibly prognosis following various interventions [14].

The depth of the natal cleft may correlate with the incidence of pilonidal disease. The severity of the condition and the outcomes of treatment may also be related to the depth of the intergluteal groove, so measuring it accurately is important.

Accurate measurement of the depth can serve several research purposes in the management of pilonidal disease. Deeper natal clefts are at higher risk of developing postoperative surgical site infections due to the cleft being prone to trapping debris and loose hairs. Coupled with moisture and low oxygen environment, these factors can be conducive to inflammation, infection and poor healing. A deeper cleft can often be harder to keep clean and dry, increasing the risk of delayed healing as well as infection. Assessing the depth can help clinicians devise and tailor postoperative wound management plans.

Overall, research into assessment of the natal cleft may allow clinicians to have a better understanding of risk factors associated with the depth of the cleft and may impact decision making by anticipating particular complications. If further research finds that a deeper cleft has a higher recurrence rate, for example, clinicians potentially may optimise their surgical approach by choosing a more appropriate technique to modify or flatten the natal cleft to reduce depth and improve postoperative outcomes.

## CONCLUSION

In conclusion, our study offers a simple and feasible technique to measure the depth of the natal cleft, with good observer agreement. We propose that future studies intent on researching the depth of the natal cleft and its relationship with severity and postoperative outcomes use this technique.

## AUTHOR CONTRIBUTIONS

**Dotun Ojo:** Conceptualization; data curation; investigation; writing – original draft; methodology. **Paul Bassett:** Methodology; validation; formal analysis. **Gregory Thomas:** Investigation; writing – review and editing; supervision. **Asha Senapati:** Conceptualization; data curation; methodology; investigation; supervision; writing – review and editing.

## FUNDING INFORMATION

We can confirm that there is no funding to declare.

## CONFLICT OF INTEREST STATEMENT

We can confirm there are no conflicts of interest to declare.

## ETHICS STATEMENT

Ethical approval for this study was obtained from National Research Ethics Committee (West of Scotland Research Ethics Service, IRAS ID 291048).

## Data Availability

The data that support the findings of this study are available on request from the corresponding author. The data are not publicly available due to privacy or ethical restrictions.

## References

[1] Milone M , Gallo G , Grossi U , Pelizzo P , D'Amore A , Manigrasso M , et al. Endoscopic sinusectomy: ‘a rose by any other name’. A systematic review of different endoscopic procedures to treat pilonidal disease. Color Dis. 2023;25(2):177–190. 10.1111/codi.16361 36217688

[2] Sondenaa K , Andersen E , Nesvik I , Soreide JA . Patient characteristics and symptoms in chronic pilonidal sinus disease. Int J Color Dis. 1995;10(1):39–42. 10.1007/BF00337585 7745322

[3] Akinci OF , Kurt M , Terzi A , Atak I , Subasi IE , Akbilgic O . Natal cleft deeper in patients with pilonidal sinus. Dis Colon Rectum. 2009;52(5):1000–1002. 10.1007/DCR.0b013e31819f6189 19502869

[4] Karydakis GE . Easy and successful treatment of pilonidal sinus after explanation of its causative process. Aust N Z J Surg. 1992;62(5):385–389. 10.1111/j.1445-2197.1992.tb07208.x 1575660

[5] Immerman SC . Patient satisfaction after the cleft‐lift procedure. Cureus. 2021;3:e17686. 10.7759/cureus.17686 PMC8489255 34650861

[6] Iesalnieks I , Schlitt HJ , Zulke C . Karydakis flap for recurrent pilonidal disease. Color Dis. 2010;12(Suppl. 3):50. 10.1111/j.1463-1318.2010.02367.x 23435676

[7] Ray K , Albendary M , Baig MK , Swaminathan C , Sains P , Sajid MS . Limberg flap for the management of pilonidal sinus reduces disease recurrence compared to Karydakis and Bascom procedure: a systematic review and meta‐analysis of randomized controlled trials. Minerva Chir. 2020;75(5):355–364. 10.23736/S0026-4733.20.08362-5 32975384

[8] Thompson MR , Senapati A , Kitchen P . Simple day‐case surgery for pilonidal sinus disease. Br J Surg. 2011;98(2):198–209. 10.1002/bjs.7292 21125608

[9] Stauffer VK , Luedi MM , Kauf PM , Schmid M , Diekmann K , Wieferich B , et al. Common surgical procedures in pilonidal sinus disease: a meta‐analysis, merged data analysis, and comprehensive study on recurrence. Sci Rep. 2018;8(1):1–28. 10.1038/s41598-018-20143-4 29449548 PMC5814421

[10] Marzouk DM , Abou‐Zeid AA , Antoniou A , Haji A , Benziger H . Sinus excision, release of coccycutaneous attachments and dermal‐subcuticular closure (XRD procedure): a novel technique in flattening the natal cleft in pilonidal sinus treatment. Ann R Coll Surg Engl. 2008;90(5):371–376. 10.1308/003588408X285955 18634729 PMC2645736

[11] Brown SR , Hind D , Strong E , Bradburn M , Din F , Lee E , et al. Real‐world practice and outcomes in pilonidal surgery: pilonidal sinus treatment studying the options (PITSTOP) cohort. Br J Surg. 2024;111(3):znae009. 10.1093/bjs/znae009 38488204 PMC10941257

[12] Lee MJ , Strong EB , Lund J , Hind D , Brown SR . A survey of treatment preferences of UK surgeons in the treatment of pilonidal sinus disease. Color Dis. 2023;25(10):2010–2016. 10.1111/codi.16696 37583061

[13] Kasim K , Abdlhamid NM , Badwan BR , Allowbany A . Is there a relation between natal cleft depth and post‐operative morbidity after different methods of excision of sacro‐coccygeal pilonidal sinus? Indian J Surg. 2012;77(S2):201–205. 10.1007/s12262-012-0762-7 26729993 PMC4692939

[14] Lee MJ , Lee E , Bradburn MD , Hind E , Strong F , Din A , et al. Classification and stratification in pilonidal sinus disease: findings from the PITSTOP cohort. Color Dis. 2024;27:e16989. 10.1111/codi.16989271 PMC11683182 38644667

